# Income Inequality, Socioeconomic Deprivation and Depressive Symptoms among Older Adults in Mexico

**DOI:** 10.1371/journal.pone.0108127

**Published:** 2014-09-24

**Authors:** Julián Alfredo Fernández-Niño, Betty Soledad Manrique-Espinoza, Ietza Bojorquez-Chapela, Aarón Salinas-Rodríguez

**Affiliations:** 1 Center for Evaluation Research and Surveys, National Institute of Public Health, Cuernavaca, Morelos, Mexico; 2 Department of Population Studies, El Colegio de la Frontera Norte, Tijuana, Baja California, Mexico; Federal University of Rio de Janeiro, Brazil

## Abstract

**Objective:**

Depression is the second most common mental disorder in older adults (OA) worldwide. The ways in which depression is influenced by the social determinants of health – specifically, by socioeconomic deprivation, income inequality and social capital - have been analyzed with only partially conclusive results thus far. The objective of our study was to estimate the association of income inequality and socioeconomic deprivation at the locality, municipal and state levels with the prevalence of depressive symptoms among OA in Mexico.

**Methods:**

Cross-sectional study based on a nationally representative sample of 8,874 OA aged 60 and over. We applied the brief seven-item version of the Center for Epidemiologic Studies Depression Scale (CES-D) to determine the presence of depressive symptoms. Additionally, to select the principal context variables, we used the Deprivation Index of the National Population Council of Mexico at the locality, municipal and state levels, and the Gini Index at the municipal and state levels. Finally, we estimated the association of income inequality and socioeconomic deprivation with the presence of depressive symptoms using a multilevel logistic regression model.

**Results:**

Socioeconomic deprivation at the locality (OR = 1.28; p<0.10) and municipal levels (OR = 1.16; p<0.01) correlated significantly with the presence of depressive symptoms, while income inequality did not.

**Conclusions:**

The results of our study confirm that the social determinants of health are relevant to the mental health of OA. Further research is required, however, to identify which are the specific socioeconomic deprivation components at the locality and municipal levels that correlate with depression in this population group.

## Introduction

The study of depression in older adults (OA) has acquired critical importance in that it ranks as the second most common mental disorder worldwide, surpassed only by dementia [1]. According to estimates, by 2030, depression will have escalated to first place in the global burden of disease, accounting for 6% of the total disability-adjusted life years [2]. Current prevalence of clinical depression among OA fluctuates between 1% and 9% [1], exerting a high impact on quality of life, perception of health, functionality, occurrence of comorbidity and economic productivity [1].

In 2005, prevalence of clinical depression in Mexico was estimated at 5% and 9.5% among men and women, respectively [3]. Alongside major clinical depression, depressive symptoms [4] have drawn considerable attention from public health initiatives. While they share a number of probable etiological explanations with clinical depression [5]–[6] and have been directly associated with mortality and suicide in this age group [6]–[7], it is estimated that their prevalence may eventually affect four times as many individuals as clinical depression [8]. In the case of Mexico, the prevalence of subclinical depression in OA over 65 years, using the Geriatric Examination for Computer Assisted Taxonomy (GMS–AGECA), was estimated at 31.9% and 32.2% in urban and rural areas, respectively, which is more than twice that the estimated prevalence using DSM-IV or ICD-10 criteria [9].

It has been amply documented that the social determinants of health (mainly contextual poverty, income inequality and social cohesion across the social organization – from the neighborhood up to the national level-) play a prominent role in a wide array of health outcomes, including maternal and child mortality, cardiovascular disease and infectious diseases [10]. However, only recently has research recognized the impact of social determinants on mental health indicators, particularly on depression [11].

According to the ecosocial theory, the health-disease process is part of a hierarchical system where causal interactions flow organically across organization levels [12] and in consequence, social influences become literally “embodied” into individuals [13]. Kim has recently adapted this model to analyze the etiology of the depression [14] by organizing the individual and contextual factors hierarchically under an explanatory model. By interweaving the two, Kim's perspective now makes it possible to understand depression as an interaction between the social context and individual susceptibility [12], [14] .

Various studies have analyzed the individual and contextual aspects of depression across a variety of OA populations, with the literature identifying the following risk factors: being female, mourning as a result of a significant life event, especially the loss of a partner, functional limitations, existence of a prior depressive episode, physical pain, presence of other pathologies, an inadequate social support network, a low educational level, living alone, social isolation, a perception of being inadequately cared for, limited access to health services and, especially, a precarious economic situation [1], [15]–[18]. On the other hand, with regard to contextual factors, it has been observed that socioeconomic disadvantage and, in general, different measures of poverty and deprivation regarding the place of residence (neighborhood, community or locality) correlate with depression in adults overall and, particularly, in OA [14].

It has been advanced that, in general, economically precarious contexts tend to generate hostile social environments (marked by insecurity and few suitable areas for recreation) [19]–[21], thus heightening exposure to everyday risks. In turn, these conditions are known to breed psychosocial stress, depleted social capital, limited access to resources and low-quality health services, all of which interact with individual susceptibility as catalysts of depression [14], [19]–[21].

Acknowledging that the different levels of contextual conditions are relevant to depression not only expands the explanatory framework for this disorder by incorporating the role of social determinants, but also provides evidence for creating interventions that transcend the individual realm. The objective of this study was to estimate the association of income inequality and socioeconomic deprivation at place of residence with the occurrence of depressive symptoms in OA aged 60 and over in Mexico.

## Methods

### Ethics statement

The sample for our work was derived from the National Health and Nutrition Survey (ENSANUT -for its initials in Spanish). This survey was approved by the Ethics and Research Committees of the National Institute of Public Health. All subjects provided written informed consent.

### Sample

*ENSANUT* 2012 was a probabilistic multi-phase survey conducted in Mexico in 2012 with national and state-level representativeness and a geographic breakdown by urban-rural strata. The total sample consisted of 55,008 households throughout Mexico, with only one adult aged 20 and over selected from each household to respond to an individual questionnaire. Out of the total 46,277 adults in this age bracket, 8,874 OA ages 60 and over constituted the analytical sample of our study. The survey design was developed to obtained representativeness for this age group at the national level. Therefore, this sample is representative of the overall older adult population in Mexico. A detailed description of the methodology and design of ENSANUT-2012 has been published elsewhere [22].

### Data collection instruments

*ENSANUT* 2012 was based on two key components: nutrition and health. The latter was probed with three individual-level questionnaires involving: age groups (children aged 9 and under, adolescents from 10 to 19, and adults aged 20 and over), health service utilization, and households. We extracted the data for our study from the individual-level questionnaire for adults aged 20 and over, and the household questionnaire. The first included the following sections: sociodemographic characteristics, self-esteem and satisfaction, overweight and obesity, principal morbidities (high blood pressure, diabetes, cardiovascular disease and hypercholesterolemia), family health history, reproductive health, immunization, preventive programs, accidents, aggression and violence, and risk factors. Additionally, this questionnaire included a module for OA aged 60 and over with the following sections: cognitive status, sight and hearing impairments, functionality, depression and falls [22]. On the other hand, the household questionnaire explored sociodemographic characteristics of all household members, health service utilization, physical housing characteristics, assets and goods, expenditures, and social protection in health.

### Variables

#### Outcome: Depressive symptoms

To measure this variable, we used the brief seven-item version of the Center for Epidemiologic Studies Depression Scale [CES-D]. The Scale covers five dimensions: dysphoric mood, motivation, concentration, loss of pleasure, and sleep disturbance [23] and it has been previously validated in Mexico [24]. The presence of clinically significant depressive symptoms was operationalized according to the total CES-D score, and a dichotomous variable was constructed where 1 (CES-D≥5) indicates the presence of significant symptoms [24].

Context variables: As principal exposure variables, we considered income inequality and socioeconomic deprivation in the localities, municipalities and states where OA resided.

#### Income inequality

We quantified this variable using the Gini coefficient derived from the Lorenz curves [25]. As a measure of statistical dispersion for income distribution of a nation's residents, the coefficient is expressed as a number between 0 and 1, where 0 indicates perfect equality (everyone receives equal incomes), and 1, total inequality (one person receives all the incomes while the others receive none) [25]. We constructed the Gini coefficient with data from the Population and Housing Census 2010 enlarged sample, which is representative of the 2,456 municipalities in Mexico [26]. The extended questionnaire used in this sample contained the following sections: fertility, child mortality, migration, indigenous language, disability, education, health services, marital status, housing characteristics and public services, household members, and economic characteristics. With regard to the last section, respondents were asked about their total household income in the previous month, in Mexican pesos. This household-specific variable served to construct the Gini coefficient for each municipality and state in Mexico using the *ineqdeco* routine developed by Jenkins for STATA 12 [27], and in which is used the following expression:
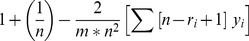



where *n* is the sample size for each group, *m* the arithmetic mean of incomes, *r_i_* the rank-ordered income values for each household, and *y_i_* the income value for each household.

Inequality indices estimated by this method have been widely used in the literature [28]. Sen [29] and Jenkins [30] provide some empirical illustrations with this same approach.

#### Socioeconomic deprivation

To measure this variable, we used the Mexican National Population Council (*CONAPO* for its initials in Spanish) Deprivation Index adjusted to 2010. Broadly validated and utilized in Mexico [31], this measurement groups a series of socioeconomic indicators (eight under a locality index and nine under a municipal and state index) into four major socioeconomic deprivation dimensions (plus a rurality indicator for municipalities and states). These, in turn, are estimated in three levels of geographic aggregation: by locality, municipal and state. The locality dimensions are: education (percentage of illiterate individuals aged 15 and above who lack elementary school), household (percentage of households with any level of overcrowding, earth floors, and no drainage, sanitary services, piped water or electricity), and work-related income (percentage of individuals earning less than two minimum wages, which equaled approximately 8.67 USD per month in 2010). The municipal and state index also includes a rurality indicator reflecting the percentage of individuals dwelling in localities with fewer than 5,000 inhabitants. Constructed using the principal component analysis, the municipal and state index allows identifying the intensity of deprivation and social exclusion by geographic area, specifically with regard to localities and municipalities/states [32]. The index is continuous, where the higher values denote greater socioeconomic deprivation.

Covariates: We included both -individual and household- specific variables identified by the literature as key factors associated with the occurrence of depressive symptoms in the OA population.

#### Individual covariates

These included: age, gender, civil status (without a partner = 1, married/cohabited =  0), paid job, schooling (years of formal education), difficulty in performing at least one of the basic activities of daily living under the Katz scale [33], difficulty in performing at least one of the instrumental activities under the Lawton scale [34], number of chronic illnesses (diabetes, high blood pressure, hypercholesterolemia, cardiovascular disease, depression, stroke and cancer), participation in household decision-making, history of any type of physical violence (broadly defined as self-reported health damage resulting from theft, aggression or battery of any type) and accidents (broadly defined as self-reported health damage resulting from an accident of any type in the last 12 months).

#### Household covariates

To measure the household socioeconomic level, we constructed an asset index with a total of 16 dichotomous [yes/no] variables related to a set of housing characteristics and the ownership of household assets. This index has been widely referred to in the literature and has been validated across a variety of contexts [35]–[37]. We built our index using a polychoric correlation matrix and the principal component analysis. The first component explained 54% of observed variance in data. The asset index was continuous, with the higher values denoting higher socioeconomic levels. We also included data on OA living arrangements using a dichotomous variable as to whether or not the OA lived alone [1 = yes].

### Statistical Analysis

We used exploratory analysis techniques, including graphics (histograms and dispersion/correlation diagrams) and descriptive statistics (means and variances), to examine properties and distributions of outcome and contextual variables. Furthermore, we applied the Mann-Whitney U and chi-square tests to analyze the bivariate association between the outcome and the independent variables (continuous and categorical, respectively). Specifically, during the exploratory analysis, we tested the linearity between the outcome and continuous contextual variables.

During the statistical analysis, we also explored the pertinence of considering the data aggregated by locality, municipality and state. For this purpose, we estimated the Intraclass Correlation Coefficient (ICC) for a three-level model [38]. ICC has two main interpretations: first, the extent to which the explained percentage of variance in the outcome variable was attributable to the different aggregation levels; and second, the extent to which individual-level observations are correlated within each aggregation level. If the observations were independent (ICC = 0), nothing would be gained by incorporating different aggregation levels. Given that the outcome variable was dichotomous, we used a multilevel logistical regression model to analyze its association with the contextual variables (income inequality and socioeconomic deprivation), controlling for covariates. This allowed us to identify any relationships within the different aggregation levels which would otherwise have been biased under other statistical techniques ignoring the hierarchical nature of data. Moreover, unlike other approaches, the multilevel analysis estimates not only the relationship of each variable to the most basic aggregation level, but also the strength of the association among variables at different levels by using terms of interaction among the different levels [38].

We adjusted three consecutive models to analyze the contribution of each aggregate level: (1) one with a random intercept at the locality level; (2) other with random intercepts for localities and municipalities, and (3) the third with random intercepts for localities, municipalities and states. We also compared the performance of the different models using the log-likelihood value, the likelihood ratio test (LRT), and the Akaike Information Criterion [AIC] [39]. Finally, we explored the possibility of having models with random slopes for the continuous contextual variables, and we used the LTR to compare these last models with the first that apply just random intercepts [38]. We used the STATA 12 program (Stata Corporation, College Station, TX, USA) for all our statistical analyses.

## Results

Of the 8,874 OA who participated in *ENSANUT* 2012, we excluded those who were unable to respond to the interview personally. The principal reasons for failure to respond were hearing or speech impairment (694 OA) and memory loss (320 OA). The final analytical sample thus consisted of 7,867 OA, of whom 53.4% were female, with an average age of 69.3 years (standard error (S.E).  =  0.15) and 6.1 years of formal education (S.E.  =  0.11). Finally, 22.3% had difficulty performing at least one of the basic activities of daily living and 18.0% at least one of the instrumental activities.

The prevalence of depressive symptoms was 35.6% (CI95%: 33.8%–37.4%). Table 1 displays the characteristics of the sample according to the presence of depressive symptoms. The following variables showed a significant association with depressive symptoms: female, absence of an intimate partner, paid job, few years of formal education, difficulty in performing basic or instrumental activities of daily living, presence of comorbidity, not being consulted about household decisions, occurrence of violent incidents or accidents in the previous 12 months, living alone, and a low household socioeconomic status (p<0.05). The bivariate analysis yielded no statistically significant relationship among the different income inequality levels. The estimated ICC values were: 6.3% (CI95%: 3.9%–9.6%) at the locality level; 4.0% (CI95%: 2.0%–7.7%) at the municipal level, and 1.5% (CI95%: 0.07%–3.3%) at the state level.

**Table 1:** Sociodemographic characteristics according to the presence of depressive symptoms in the study sample.‡

	Depressive symptoms		
	No (n = 5,040)	Yes (n = 2,827)	p-value†
Individual			
Age (years)	69.27(0.19)	69.24(0.23)	n.s
Gender (Female)	0.49	0.60	***
No current intimate partner	0.35	0.42	***
Paid work	0.29	0.25	**
Years of schooling	6.42(0.15)	5.53(0.14)	***
Difficulty in performing basic activities of daily living	0.13	0.18	***
Difficulty in performing instrumental activities	0.13	0.26	***
No. of chronic illnesses	0.88(0.03)	1.14(0.03)	***
Participation in household decision-making (1 = no).	0.06	0.12	***
Accident in the last 12 months (1 = yes)	0.06	0.09	***
Violence in the last 12 months (1 = yes)	0.01	0.03	***
Household			
Asset index	−0.04 (0.02)	−0.40 (0.03)	***
Older adult lives alone	0.23	0.26	***
Locality			
Deprivation Index	−1.00(0.01)	−0.93(0.01)	***
Municipality			
Deprivation Index	−0.93(0.01)	−0.78(0.02)	***
Income inequality (Gini coefficient)	0.45(0.01)	0.45(0.01)	n.s
State			
Deprivation Index	0.01(0.01)	0.11(0.02)	***
Income inequality (Gini coefficient)	0.45(0.01)	0.45(0.01)	n.s

‡ Share or mean (standard error].

† p values under the chi-square and Mann-Whitney U tests..

*p<0.10; **p<0.05; ***p<0.01.

n.s.: non-significant.

Table 2 presents the results of the multilevel logistic regression models. As the exploratory analysis indicated that the household asset and locality deprivation indices were not linearly associated with to the logarithm of odds on the presence of depressive symptoms, both variables were incorporated as tertiles in the regression models. Additionally, we adjusted all models using the locality and municipality population sizes as well as to the percentage of rural population at both levels.

**Table 2:** Multilevel logistical regression models for the association between the presence of depressive symptoms and contextual variables: income inequality and socioeconomic deprivation^‡^.

Variables	Model 1			Model 2			Model 3		
	OR†	CI 95%	p	OR	CI 95%	p	OR	CI 95%	p
***Individual***									
Age (years)	1.00	0.99–1.00	n.s	0.99	0.99–1.00	n.s	0.99	0.99–1.00	n.s
Female gender	1.38	1.23–1.55	***	1.38	1.23–1.54	***	1.37	1.23–1.54	***
No intimate partner	1.16	1.02–1.31	**	1.16	1.02–1.32	**	1.16	1.03–1.32	**
Paid work	0.94	0.83–1.06	n.s	0.94	0.83–1.06	n.s	0.93	0.82–1.06	n.s
Not consulted in household decision-making	1.66	1.39–1.99	***	1.65	1.38–1.98	***	1.64	1.37–1.97	***
Years of Schooling	0.97	0.95–0.98	***	0.97	0.95–0.98	***	0.97	0.95–0.98	***
Number of illnesses	1.32	1.25–1.39	***	1.32	1.26–1.38	***	1.32	1.25–1.38	***
Difficulty in performing instrumental activities	1.45	1.26–1.67	***	1.46	1.26-1-68	***	1.45	1.26–1.68	***
Difficulty in performing basic activities	1.85	1.63–2.11	***	1.85	1.62–2.10	***	1.85	1.62–2.10	***
Accident in the previous 12 months	1.50	1.24–1.82	***	1.50	1.24–1.82	***	1.53	1.26–1.85	***
Violence in the previous 12 months	2.40	1.56–3.67	***	2.30	1.56–3.67	***	2.41	1.57–3.69	***
***Household***									
Asset index (Reference tercile 1]									
Tercile 2	0.87	0.78–0.97	**	0.87	0.77–0.97	**	0.86	0.77–0.97	**
Tercile 3	0.80	0.67–0.95	**	0.79	0.67–0.95	**	0.79	0.66–0.94	***
Older adult living alone	1.09	0.95–1.26	n.s	1.10	0.95–1.26	n.s	1.10	0.96–1.27	n.s
***+ Locality***									
Rurality	1.10	0.89–1.36	n.s	1.08	0.88–1.33	n.s	1.06	0.86–1.31	n.s
Deprivation index (Reference tercile 1]									
Tercile 2	1.19	0.98–1.45	*	1.18	0.97–1.44	*	1.15	0.94–1.40	n.s
Tercile 3	1.33	1.02–1.73	**	1.31	1.00–1.70	**	1.28	0.98–1.67	*
***Municipality***									
Income inequality (Gini Coefficient)	1.68	0.40–7.03	n.s	1.72	0.40–7.52	n.s	1.68	0.37–7.30	n.s
Deprivation index	1.17	1.05–1.30	***	1.16	1.04–1.30	***	1.16	1.04–1.30	***
**State level**									
Income inequality (Gini coefficient)	0.41	0.03–4.88	n.s	0.49	0.04–6.54	n.s	0.45	0.01–16.14	n.s
Deprivation Index	1.01	0.93–1.09	n.s	1.01	0.93–1.09	n.s	1.00	0.91–1.11	n.s
**Variance components**									
	**Variance**	**SE**		**Variance**	**SE**		**Variance**	**SE**	
Locality	0.097	0.037		0.023	0.047		0.021	0.046	
Municipality				0.075	0.038		0.054	0.036	
State level							0.026	0.013	
									
Log-likelihood value	−4745.401			−4743.051			−4738.582		
LRT				4.70 (p<0.05]¥			8.94 (p<0.01]£		
AIC	9542.802			9540.103			9533.164		

‡ Model 1: Random intercept at locality level; random intercepts for localities and municipalities; random intercepts for localities, municipalities and states.

† OR  =  Odds ratio; CI 95%: 95% Confidence Interval.

¥ Model 2 versus Model 1.

£ Model 3 versus Model 2.

*p<0.10; **p<0.05; ***p<0.01.

LRT: Likelihood Ratio Test.

AIC: Aikaike Information Criterion.

n.s.: non-significant.

+Mexico is organized politically and administratively into states, which are composed of municipalities, the smallest administrative units in the country. In turn, municipalities are composed of localities, which are defined as “any place occupied by one or more buildings utilized as housing - inhabited or not – and which are designated by a name recognized by law or custom.” Rural localities are those with less than 2500 inhabitants and which are not municipal centers. Source: National Geostatistical Framework 2010. National Institute of Statistics, Geography and Data Processing.

In general, the association of the outcome variable with the individual and household variables was consistent across estimated models, in particular with regard to the following variables: female, absence of an intimate partner, not being consulted on household decisions, few years of schooling, difficulty in performing the basic or instrumental activities of daily living, presence of comorbidities and the tertiles of asset index. In comparing the three estimated models, the third (with random intercepts for the three levels) demonstrated the best fit, not only by the log-likelihood and AIC values, but also and most importantly, by the LRT results.

The third model showed a statistically significant association between the presence of depressive symptoms and the following individual variables: female (OR = 1.37; CI95%:1.23–1.54), absence of an intimate partner (OR = 1.16; CI95%; 1.03–1.32), not being consulted about household decisions (OR = 1.64; CI95%: 1.37–1.97), years of schooling (OR = 0.97; CI95%: 0.95–0.98), presence of multimorbidity (OR = 1.32; CI95%: 1.25–1.38), difficulty in performing at least one instrumental activity of daily living (OR = 1.45; CI95%: 1.26–1.68), difficulty in performing at least one basic activity of daily living (OR = 1.85; CI95%: 1.62–2.10) and self-reported accidents (OR = 1.53; CI95%: 1.26–1.85) or violent incident(s) (OR = 2.41; CI95%: 1.57–3.69) during the previous 12 months. Finally, at the household level, we observed a significant and protective association between the outcome variable and a higher socioeconomic status (OR = 0.79; CI95%: 0.66–0.94, when comparing tertiles 1 and 3).

Regarding the association of the contextual variables with the presence of depressive symptoms, on comparing tertiles 3 versus 1, the association of socioeconomic deprivation index was marginally significant at the locality level (OR = 1.28; p<0.10), and significant at the municipality level (OR = 1.16, p<0.01). While income inequality showed no association at any of the aggregation levels analyzed (locality, municipality or state). Furthermore, no evidence was found to support the use of random slopes at either the municipality (LRT = 0.10; p = 0.99) or the state (LRT = 0.22; p = 0.89) levels.

## Discussion

In line with other recent studies on OA, we found significant association between the presence of depressive symptoms and a number of variables at the individual level, in particular: female [17], [40]–[43], absence of an intimate partner [17], [41]–[43], a low educational level or illiteracy [17], [41]–[42], lack of empowerment in household decision-making (especially with regard to financial matters) [43]–[44], disability or functional limitations [40]–[41], presence of comorbidity [40]–[43], and chronic exposure to psychosocial stress [42]. The latter could be an important factor in the strong connection we found between depressive symptoms and self-reported incidents of physical violence or accidents in the previous 12 months. This is consistent with a recent Brazilian study [45], which found a strong association between exposure to violent, traumatic events and the prevalence of various psychiatric disorders, including major clinical depression. Other studies have established an association between a low socioeconomic status at either the individual or household level and depression among OA [15]–[17], [43]–[48].

Our principal finding was a strong link between the presence of depressive symptoms and socioeconomic deprivation at both the locality and municipality levels. Other studies have reported such a link at even more immediate aggregation levels, namely, the neighborhood [49]–[55]. In this regard, a recent theoretical framework for exploring the role of the social context has suggested that the highly adverse social conditions in the areas immersed in steepest socioeconomic deprivation –specifically, crime and restricted access to resources of all sorts, such as nutritional diversity, an adequate physical environment and health care– erode interpersonal relationships and social capital, interacting with individual susceptibility to heighten the risk of depression [14]. This explanatory framework could well apply to higher-level conditions, such as those characterizing the localities [43] and municipalities inhabited by our sample of OA.

The link between socioeconomic deprivation at the locality level and the presence of depressive symptoms in Mexico has already been described. OA living in deprived environments routinely confront more adverse conditions leading to feelings of impotence and hopelessness, both, recognized factors in depression [43]. Multilevel analyses in Belgium [56] and Japan [57] have reported connections between socioeconomic deprivation and depression in OA at the municipality level, while another work has correlated poverty and depression in the United States at the county level [58]. While clearly recognizing the importance of the individual-level factors identified in this study, we emphasize the need to comprehend the impact of socioeconomic disadvantages at place of residence as a critical social determinant of depression in OA.

The association found by other studies between income inequality and the presence of depressive symptoms at the state level was not encountered in our work [59]. The failure of this and other studies to find this association may stem from the fact that income inequality and socioeconomic deprivation have separate causal pathways. While socioeconomic deprivation mainly refers to the material conditions of the environment and access to various social services, income inequality is a more complex social determinant because it refers to not only the deficit but also the social distribution of available resources and the social consequences of the inequality [59].

In consequence, the effect of the first determinant would be more direct, as suggested in the Kim model [14]; while, in contrast, the effect of socioeconomic marginalization on mental health could be explained by more indirect etiologies, such as the ecological effects of social disorganization [59]. In one of the most explored theoretical models, inequality generates social corrosion, corruption and alterations in social capital, thereby creating a hostile social environment, which produces a risk to overall health, including mental health [60]. However, these pathways have not been fully elucidated and, therefore, this constitutes a promising field of research. In addition, its associations reported for this determinant vary by level and tend to be particularly salient at the aggregation levels most proximate to the event being studied.

The findings of this work can be clearly understood under the theory of the social determinants of health, particularly under the ecosocial theory [12]. The social context - measured in our study by socioeconomic deprivation at the locality and municipal levels - is a fundamental factor in the incidence of numerous chronic illnesses, such as cardiovascular disease, obesity, cancer and infant mortality [19], [61]. In our work, locality and municipal level deprivation is a multidimensional construct indicative of restricted access to essential public services, such as education, housing and income, which, in turn, can be important determinants of health outcomes. Disaggregating the potential impact of the deprivation index on the presence of depressive symptoms in OA at the municipal level would probably provide more conclusive data, given that, as a summary indicator, the deprivation index simultaneously reflects the combined association of numerous interrelated social determinants –social capital, physical environment and violence - that are also known to exert an impact on depressive symptoms [40], [43], [49]. In fact, alternative contextual variables incorporated into our secondary analyses –homicide rates, levels of health coverage, criminality indicators, and government spending at the municipality and locality levels– revealed no significant association with the presence of depressive symptoms. On the other hand, the multidimensional character of the index may precisely constitute its strength, since it expands the conceptual horizon to encompass a host of interlocking associations, including that of depression and socioeconomic deprivation at the locality and municipal levels.

The results of our study suggest that the current public health approach to depression needs to be expanded. Policy makers might consider that transforming the social environment and generating economic development in the poorest areas could be important strategies that would have both a multidimensional impact on health promotion and protect OA from the social risks to mental health. Additionally, providing some resources in the immediate environment– such as access to health services and recreational and social support – would strengthen the individual resilience of OA to mental disorders [14].

This study has several limitations. One is related with its cross-sectional design, which does not permit drawing firm conclusion about causal associations. Our findings are thus limited to suggesting potential connections requiring more in-depth analysis in subsequent studies. Another limitation derives from the different timing of the various measurements, with the contextual and individual/household measurements conducted in 2010 and 2012, respectively. Nonetheless, it can be expected that, on the one hand, relative differences in the deprivation index would hold for two years, and most importantly, on the other, a potential effect at the municipal level would correlate more with prolonged exposure to socioeconomic deprivation than with a simultaneous event. A final weakness in our study is the fact that, due to lack of information, we were unable to explore other important factors in OA depression, such as significant life events and social networks, which have been recognized as crucial in the etiology of depression.

## Conclusions

The results of this study suggest that deprivation at the municipal and locality levels is associated with depressive symptoms, thus inviting proposals for comprehensive public policy actions that view the problem of depression in older adults also as an expression or symptom of more generalized social malaise. Evidence on the role of socioeconomic deprivation will gain scientific and political importance only to the extent that epidemiologic findings translate into a transformation of the social systems [62]. Recognizing that poverty at the municipal and locality levels impacts the health of older adults should galvanize our society to undertake the profound changes required in the social structure.
